# Editorial: Hyperglycemia and Coronary Artery Diseases: Physio-Pathological Findings and Therapeutic Implications

**DOI:** 10.3389/fphar.2022.901815

**Published:** 2022-05-19

**Authors:** Raffaele Marfella, Massimo Federici, Giuseppe Paolisso

**Affiliations:** ^1^ Department of Advanced Medical and Surgical Sciences, University of Campania Luigi Vanvitelli, Naples, Italy; ^2^ Mediterraneo Cardiocentro, Napoli, Italy; ^3^ Department of Systems Medicine, University of Rome Tor Vergata, Rome, Italy

**Keywords:** atheroclerosis, hyperglycemia, coronary artery diseas, macro-angiopathy, type 2 diabetes

Many observational studies have documented that hyperglycemia frequently occurs among patients hospitalized with the acute coronary syndrome (ACS) and without diabetes mellitus (Capes et al., 2000; Kosiborod et al., 2005; Ferreira et al., 2021). Epidemiological studies showed that 25–50% of ACS patients had elevated blood glucose levels at admission (Deedwania et al., 2008). Why are we concerned by these observations? Because several studies have shown that acute hyperglycemia at admission (AH) is independently associated with a poor early and late prognosis in ACS patients, especially those diagnosed with acute myocardial infarction (AMI) (Deedwania et al., 2008). In particular, high glucose levels at admission increased the intra-hospital mortality by twice compared to normoglycemic diabetic patients and 3.9-fold vs. normoglycemic patients without diabetes (Capes et al., 2000). Thus, AH in ACS is an independent risk factor for cardiovascular mortality, especially in patients without known diabetes. Which blood glucose levels affect the ACS outcomes? To date, despite numerous studies regarding hyperglycemia during cardiovascular events have been published, there’s not a clear definition for AH in the setting of ACS. Most early studies defined hyperglycemia by the first available glucose value or admission blood glucose levels (Capes et al., 2000; Kosiborod et al., 2005; Deedwania et al., 2008; Ferreira et al., 2021). Nevertheless, the cutpoint of AH used to define hyperglycemia in patients with ACS was different from study to study. However, the most acceptable description of AH refers to the first acquired blood glucose within 24 h of admission (Kojima et al., 2020). Back in 2008, the American Heart Association Scientific (AHA) Statement on Hyperglycemia and Acute Coronary Syndrome suggested using an ABG level >140 mg/dL as the definition of hyperglycemia under such circumstances irrespective of fasting status (Deedwania et al., 2008). Therefore, it is essential to know what occurs in the heart when blood glucose rises to more than 140 mg/dl during ACS. In this context, hyperglycemia may exacerbate acute cardiac disease in various ways, including compounding microvascular obstruction (Sardu et al., 2019a), attenuating endothelium-dependent vasodilation (D'Onofrio et al., 2016), impairing platelet nitric oxide responsiveness and endothelial repair (Marchetti et al., 2006; Balestrieri et al., 2013), increasing coronary thrombosis (Menghini et al., 2014), and promoting direct cardiomyocyte damage (D'Onofrio et al., 2020). Thus, hyperglycemia may be responsible for both coronary and cardiomyocytes impairments. The inflammatory burden in the peri-infarct region is associated with worse short- and mid-term outcomes because the inflammatory response in this region probably may amplify myocardial necrosis. In this context, hyperglycemic stress during ACS is associated with increased levels of some inflammatory markers, including C-reactive protein and interleukin-18, and enhanced expression of natural killer cells (CD16/CD56) associated with reduced expression of some T cells (CD152) known to limit the immune process in patients presenting with ACS (Marfella et al., 2013). These results fit with animal studies showing increased levels of proinflammatory cytokines (tumor necrosis factor-α, interleukin-6, interleukin-18) and peroxynitrite (an index of oxidative stress) in the heart tissue of hyperglycemic mice (Marfella et al., 2012). Another study (Marfella et al., 2004a) observed that the glucose levels correlated strictly with myocardial apoptosis and greater infarct size and a reduced expression of some critical angiogenic factors, such as hypoxia-inducible factor-1α, and vascular endothelial growth factor with nondiabetic patients with ischemia (Paolisso et al., 2021). Therefore, hyperglycemia may lead to reduced angiogenesis during myocardial ischemia and acute myocardial infarction, affecting the regenerative potential of the myocardium during acute infarction (Marfella et al., 2004a). Thus, altering inflammatory and oxidative stress in myocardial acute infarcted tissue, hyperglycemia may lead to an abnormal myocardial damage extension size (Paolisso et al., 2020). Although AH is an independent predictor of short- and long-term mortality in patients with and without diabetes, therapeutic strategies to improve the CV outcomes in this high-risk population are lacking. In this context, the primary percutaneous coronary intervention (PPCI) as the mainstay of treatment in patients with ST-segment elevation myocardial infarction (STEMI) (Marfella et al., 2004b; Paolisso et al., 2020) appears to be less effective in hyperglycaemic patients (Marfella et al., 2004b). The incidence of restenosis (Marfella et al., 2013), heart failure, re-infarction, and death in hyperglycemic STEMI patients are more common than the normoglycemic ACS patients and significantly reduce the effectiveness of PPCI. To reduce the increased mortality from AH, the AHA consensus suggests insulin protocols to normalize glucose levels during ACS (Deedwania et al., 2008). However, treatment with insulin has shown contradictory results. Despite data from monocentric studies, non-randomized clinical trials, and a meta-analysis suggesting the benefits of treatment with insulin, larger randomized control trials have failed to confirm improved survival (Vergès et al., 2012; Singh et al., 2015; He et al., 2022). Therefore, the hyperglycemia treatment during STEMI remains unclear. Because hyperglycemia causes overproduction of reactive oxygen species and inflammation from thrombus plaque, favoring athero-thrombotic embolization and poor myocardial infarction outcomes reducing epicardial blood flow (Jacobi et al., 2012), thrombus aspiration (TA) during primary PPCI may be an effective method for reducing distal embolization and improving microvascular perfusion in hyperglycemic ACS patients (Corbett, 2012). However, studies now firmly point to no clinical benefit of routinely using adjunctive TA in treating STEMI patients undergoing PPCI in the general population (Ikari et al., 2008; Lagerqvist et al., 2014; Tilsted and Olivecrona, 2015; Jolly et al., 2016; Sardu et al., 2018; Sardu et al., 2019b). Nevertheless, recent studies have been evidenced that beyond the early restoration of epicardial blood flow, TA during PPCI, limiting inflammatory distal embolization, and preserving microcirculatory integrity may have a central role in the management of hyperglycemic STEMI patients (Corbett, 2012; Jacobi et al., 2012). In the meantime, the question is whether the mere quantitative evaluation of thrombus burden is sufficient to individualize subjects who can benefit from TA in high-risk patients as hyperglycemic STEMI patients.
